# Correction to: Disinformation in Open Online Media

**DOI:** 10.1007/978-3-030-61841-4_19

**Published:** 2020-10-19

**Authors:** Max van Duijn, Mike Preuss, Viktoria Spaiser, Frank Takes, Suzan Verberne

**Affiliations:** 8 Leiden Institute of Advanced Computer Science, Leiden University, Leiden, The Netherlands; 9 Leiden Institute of Advanced Computer Science, Leiden University, Leiden, The Netherlands; 10 School of Politics and International Studies, University of Leeds, Leeds, UK; 11 Leiden Institute of Advanced Computer Science, Leiden University, Leiden, The Netherlands; 12 Leiden Institute of Advanced Computer Science, Leiden University, Leiden, The Netherlands; 13 Leiden Institute of Advanced Computer Science, Leiden University, Leiden, The Netherlands; 14 Leiden Institute of Advanced Computer Science, Leiden University, Leiden, The Netherlands; 15 School of Politics and International Studies, University of Leeds, Leeds, UK; 16 Leiden Institute of Advanced Computer Science, Leiden University, Leiden, The Netherlands; 17 Leiden Institute of Advanced Computer Science, Leiden University, Leiden, The Netherlands

## Correction to: M. van Duijn et al. (Eds.): *Disinformation in Open Online Media*, LNCS 12259, 10.1007/978-3-030-61841-4

In the original online version of the chapter 5 was previously published non-open access. It was changed to open access retrospectively under a CC BY 4.0 license and, the presentation of Table 3 was different to that of Tables 2 and 4. This has been corrected. In addition, Tables 5 - 8 have been moved from the main text to Appendix B, at the request of the authors.

The original version of the chapter 11 contained an error in Table 2, which also affected Section 3.1 and the Conclusion. The original figure in Table 2 indicated that one community was retweeting from a smaller number of accounts than the other communities. A recalculation following publication showed that the community was retweeting from a pool of about the same number of accounts as the other communities. This has been updated.

